# Impact of fixed orthodontic retainers on oral health-related quality of life: a longitudinal prospective study

**DOI:** 10.1590/2177-6709.29.1.e242317.oar

**Published:** 2024-03-04

**Authors:** Melany Clarissa Gámez MEDINA, Cibelle Cristina Oliveira dos SANTOS, Beatriz Oliveira LIMA, Marina Bosi FERREIRA, David NORMANDO

**Affiliations:** 1 Universidade Federal do Pará, Faculdade de Odontologia, Departamento de Ortodontia (Belém/PA, Brazil).; 2 Associação Brasileira de Odontologia, Especialização em Ortodontia (Belém/PA, Brazil).

**Keywords:** Orthodontic retainers, Quality of life, Orthodontics, Contenções ortodônticas, Qualidade de vida, Ortodontia

## Abstract

**Objective:**

The aim of the present study was to assess the impact of orthodontic retainers on oral health-related quality of life (OHRQoL) in the short and long terms after orthodontic treatment.

**Methods:**

Data from 45 patients up to three years after orthodontic treatment (T0) were analyzed. Patients were reassessed four years (T1) after T0. OHRQoL was measured using the OHIP-14 (Oral Health Impact Profile-14) questionnaire. The presence of a fixed retainer in the upper and/or lower arches, sex, and age were the predictive variables evaluated at T0 and T1. The occurrence of retainer fracture at T0 was clinically evaluated. Due to the COVID-19 pandemic, clinical examination on T1 was not possible, so the OHIP-14 and the self-perception of changes in teeth position and fracture of retainers were examined using an on-line questionnaire.

**Results:**

At the initial examination, the presence of upper retainers had a negative impact on quality of life (*p*=0.018). The OHIP-14 value increased significantly from T0 to T1 (*p*=0.014), regardless of the presence of retainers. The fracture or debonding of the retainer reported by the patient was the only variable that had a negative impact on OHRQoL (*p*=0.05).

**Conclusion:**

The use of fixed upper retainers suggests a negative impact on the quality of life of the orthodontic patient after the end of orthodontic treatment. This impact, however, is negligible in the long term, except when associated with fracture or debonding. This study emphasizes the need for continuous follow-up of orthodontic patients during the retention period.

## RESUMO

**Objetivo:**

O objetivo deste estudo foi avaliar o impacto das contenções ortodônticas na qualidade de vida relacionada à saúde bucal (QVRSB) em curto e longo prazos após o tratamento ortodôntico.

**Métodos:**

Foram analisados dados de 45 pacientes até três anos após o tratamento ortodôntico (T0). Os pacientes foram reavaliados quatro anos (T1) após T0. A QVRSB foi mensurada usando o questionário OHIP-14 (*Oral Health Impact Profile*-14). A presença da contenção fixa nas arcadas superior e/ou inferior, o sexo e a idade foram as variáveis preditoras em T0 e T1. A ocorrência de quebra da contenção em T0 foi avaliada clinicamente. Em razão da pandemia da COVID-19, não foi possível o exame clínico em T1; assim, a autopercepção das alterações na posição dos dentes e a quebra ou descolagem das contenções foram registradas por meio de um questionário online.

**Resultados:**

No exame inicial, a presença da contenção superior apresentou um impacto negativo na qualidade de vida (*p*=0,018). Em T1, o valor de OHIP-14 aumentou significativamente em comparação a T0 (*p*=0,014), independentemente da presença das contenções. A quebra ou descolagem da contenção relatada pelo paciente foi a única variável que apresentou um impacto negativo na QVRSB (*p*=0,05).

**Conclusão:**

O uso de contenção fixa superior sugere um impacto inicial negativo na qualidade de vida do paciente após o fim do tratamento ortodôntico. Esse impacto, entretanto, é insignificante em longo prazo, exceto quando associado à descolagem ou quebra das contenções ortodônticas. Esse estudo enfatiza a necessidade de acompanhamento contínuo das contenções fixas após a finalização do tratamento ortodôntico.

## INTRODUCTION

Oral health-related quality of life (OHRQoL) is defined as the impact of oral conditions on an individual’s physical, emotional and social well-being.^1^ In addition to promoting an improvement in esthetics and occlusion function, orthodontic treatment is associated with a positive impact on quality of life.^2^ ^-^ ^5^

Despite the benefits of orthodontic treatment on quality of life, little has been studied about the changes after the end of treatment. In the long term, patient satisfaction is only slightly associated with the stability of orthodontic treatment, and does not seem to depend on the initial occlusal condition or the final result of the orthodontic treatment.^6^ A systematic review analyzed factors associated with patient satisfaction after orthodontic treatment and concluded that satisfaction was associated with perceived esthetic outcomes, psychological benefits and quality of care. On the other hand, dissatisfaction was associated with the duration of treatment, levels of pain and discomfort and the use of the orthodontic retainers.^7^

An orthodontic retainer, despite its negative impact on quality of life,^7^ has been used as a protocol to maintain teeth in the ideal aesthetic and functional position after orthodontic treatment.^8^ ^-^ ^10^ It presents a diversity of designs, including fixed and removable models. Fixed retainers are more commonly used, as they have better aesthetics, less need for patient cooperation, are more effective and seem more suitable for prolonged use.^11^ However, the need for a precise bonding technique, the risk of fracture and the tendency to periodontal problems - due to the difficulty imposed on oral hygiene - are some of its disadvantages.^11^ These factors can have an impact on the patient’s quality of life in the short and long terms. Among the undesirable effects related to the use of fixed retainers are the risks of debonding^12^ and, consequently, canine tipping.^13^ ^,^ ^14^ Fixed retainers bonded canine to canine seem to be effective in maintaining the alignment of the lower incisors, while in the maxilla there are doubts about the need to use retainers in the long term.^15^ ^,^ ^16^

Few studies have evaluated the quality of life related to oral health after orthodontic treatment and the impact of orthodontic retainers in this condition. From the patient’s perspective, a removable retainer made with a vacuum-pressed acetate plate, when compared to a fixed lower retainer, is associated with a higher level of pain or discomfort in the short term (2 weeks) or medium term (18 months) after treatment. In addition, patients report greater difficulty in adapting to removable retainers.^17^

Despite the literature pointing out that the presence of a retainer is capable of producing a negative impact on the quality of life immediately after installation,^7^ ^,^ ^17^ it does not appear to exist any analysis of the impact on the presence of long-term retainers. Due to the need to use retainers permanently, to avoid relapses in the long term^18^ ^,^ ^19^, the hypothesis of this study is that the presence of a long-term fixed retainer does not negatively impact quality of life related to oral health. For this reason, our aim is to report the impact of the presence of fixed retainers in the upper and lower arches on short- and long-term oral health-related quality of life after orthodontic treatment.

## MATERIAL AND METHODS

### ETHICAL CONSIDERATIONS

This longitudinal, prospective study was designed following the guidelines of the STROBE protocol for observational studies.^20^ The study was approved by the Ethics Committee for Research on Human Beings of the Hospital Ophir Loyola (HOL, Belém/PA, Brazil), under protocol number 2.254.339. The selected individuals authorized their participation by signing a Free and Informed Consent Term, in accordance with the National Health Council (CNS), resolution 466/12.

### STUDY DESIGN

Initially, data from 48 patients who had completed orthodontic treatment for at least three years were collected in three private orthodontic clinics, and analyzed (T0). The data were obtained from a questionnaire on quality of life related to oral health (T0). Patients completed this questionnaire between October and November 2016. Due to the COVID-19 pandemic, the questionnaire was completed again in February and March 2021 using the online format (Google Forms), four years later (T1) by 45 patients (93.8%).

### PARTICIPANTS

#### Inclusion criteria

Patients aged at least 18 years, of both sexes, who had completed orthodontic treatment for three years or more, only patients who attended routine post-treatment control appointments, with treated normal occlusion and clinical stability after orthodontic treatment (TO), with or without fixed retainer (3x3 type) in the upper and/or lower arches.

#### Exclusion criteria

Patients with systemic diseases, carious lesions, periodontal disease, cleft lip and palate, who underwent orthognathic surgery, were rehabilitated with prostheses or who presented any complaint of relapse of orthodontic treatment.

### VARIABLES, DATA SOURCES, AND MEASUREMENT

To assess the quality of life of patients after orthodontic treatment, the OHIP-14 (Oral Health Impact Profile-14) questionnaire was used and the final score was considered as the outcome variable. The OHIP-14 is composed of questions related to the impact of the oral health condition on well-being. The questions involve pain perception, psychosocial status, social interaction, and daily activities. The instrument version of this study was previously adapted for Brazilians,^21^ and patients were asked to answer the questionnaire based on their self-perception related to the use of orthodontic retainers. The OHIP-14 consists of 14 questions that measure oral health-related quality of life through scores (never = 0, occasionally = 1, sometimes = 2, repeatedly = 3, always = 4) and can result in a maximum of 56 points. The higher the value of the sum of the scores, the worse the patient’s quality of life.^22^ ^,^ ^23^

The presence of a fixed retainer in the upper and/or lower arches, sex, and age were the predictive variables evaluated at T0 and T1. In the evaluation performed at T0, all patients had excellent clinical stability. This was confirmed through clinical examination, in which the presence of retainers and the occurrence of fractures were measured, which was considered the independent variable at T0. Due to the COVID-19 pandemic, it was not possible to perform the clinical examination at T1. Thus, self-perception related to changes in the position of the teeth and debonding or fracture of any of the retainers was evaluated through a simple dichotomous question (yes or no) to the patient. These two measures of self-perception were included as independent variables.

### SAMPLE SIZE

The main objective of the study was to investigate whether there was a difference between the groups at T0 and T1 regarding the predictive variables in the OHIP-14 questionnaire. To determine the minimum sample size required, the probability of error was 5%, the power 80%, and the effect size was considered as 4 scores as the difference between means. Based on this information, the minimum sample size required was calculated as 40 individuals.

### STATISTICAL ANALYSIS

Poisson regression with robust variance was used to examine the association between quality of life at T0 and T1 and the predictor variables. Repeated measures ANOVA was used to assess changes in OHIP-14 from T0 to T1. Statistical analysis was performed using Jamovi v. 1.6.21.0 software (Sydney, Australia). The significance level adopted was 5%.

## RESULTS

Three of the 48 patients examined at T0 were either not found or did not respond, resulting in 45 (93.8%) patients in the final sample who responded to the questionnaire at T1.

The paired sample consisted of 20 (44.4%) male and 25 (55.6%) female individuals at times T0 and T1. At T0, the mean age was 23.6 years (19 to 51 years) for both sexes. Twenty-eight (n=28, 62.2%) patients used a fixed retainer in the upper arch, while 17 (37.8%) did not. In the lower arch, 40 (88.9%) patients used fixed retainers, while 5 (11.1%) did not. Thirteen (n=13, 28.9%) patients had some type of retainer fracture assessed from the clinical examination, 30 (66.7%) patients did not, and 2 (4.4%) were without a retainer.

At T1, the mean age was 27.8 years (22 to 56 years) for both sexes. Twenty-two (n=22, 48.9%) patients used a fixed retainer in the upper arch, while 23 (51.1%) did not. In the lower arch, 35 (77.8%) patients used fixed retainers and 10 (22.2%) did not. Regarding the self-perception of changes in the position of the teeth, 26 (57.8%) patients reported that they did not perceive any change, 18 (40%) reported some change in the position of the teeth, while 1 (2.2%) patient reported they did not know. As for self-perception of retainer fracture, 23 (51.1%) patients reported not noticing this occurrence, 13 (28.9%) patients reported some debonding or fracture in the retainer, 8 (17.8%) patients had no retainer in both arches, while 1 (2.2%) patient was unable to report accurately (Table 1).

**Table 1:** Distribution of the sample (frequency), mean and standard deviation, according to OHIP-14 at T0 and T1, age, sex, type of retainer, fracture and self-perception of changes in the position of the teeth.

	Frequency (f) / Mean		Percentage or standard deviation	
Variables	T0 (45)	T1 (45)	T0 (45)	T1 (45)
OHIP-14	6.04	8.31	5.77	7.57
Age	23.6	27.8	5.89	5.92
Sex (f)				
Male	20	20	44.4%	44.4%
Female	25	25	55.6%	55.6%
Upper retainer (f)				
Absent	17	23	37.8%	51.1%
Present	28	22	62.2%	48.9%
Lower retainer (f)				
Absent	5	10	11.1%	22.2%
Present	40	35	88.9%	77.8%
Fracture/debonding (clinically assessed at T0 and self-perception at T1)				
No	30	23	66.67	51.11%
Yes	13	13	28.89	28.89%
No retainer	2	8	4.44	17.78%
Not reported		01		2.22%
Self-perception of change in teeth position				
No	-	26	-	57.8%
Yes	-	18	-	40%
Not reported	-	01	-	2.2%

Regarding the use of a fixed upper retainer at T0, six (13%) patients stopped using it at T1. Regarding the use of the lower fixed retainer at time T0, 5 (11%) patients stopped using it at T1.

At T0, the mean of the OHIP-14 was 6.04 (SD 5.77) and no significant association was detected between the quality of life and the variables sex, age, and use of a lower fixed retainer. However, a negative impact on quality of life was observed with the presence of an upper fixed retainer (95% CI = 1.21 - 4.29, *p*= 0.018). Patients who used fixed upper retainers had an OHIP value 2.19 times higher, compared to those who did not. There was no negative impact at T0 on the OHRQoL (*p*=0.666) for 28.9% of patients with a detached or fractured retainer (Tables 1 and 2).

**Table 2:** Poisson regression analysis to assess the impact of independent variables on the quality of life at T0.

	Univariate model		
Variables	PR	95% CI	P-value
Sex			
Female - male	1.51	0.87 - 2.72	0.157
Age	1.00	0.95 - 1.05	0.883
Upper retainer			
Absent	1		
Fixed	2.19	1.21 - 4.29	0.018*
Lower retainer			
Absent	1		
Fixed	1.88	0.70- 7.35	0.283
Fracture/debonding of retainer			
Yes - No	0.87	0.44-1.60	0.666

CI = Confidence Interval, * p < 0.05, PR = Prevalence Ratio.

The regression analysis at T1 showed no significant association between quality of life and the variables age, sex, presence or absence of upper and/or lower fixed retainer, and self-perception related to the change in the position of the teeth. However, a negative impact on quality of life was observed associated with the report of fracture and/or debonding of the retainer (95% CI = 1.01 - 3.07, *p*=0.05) in the multivariate analysis. Patients who reported debonding or fracture in the retainer had an OHIP value 1.76 times higher, compared to those who did not (Table 3).

**Table 3:** Poisson regression analysis to assess the impact of independent variables on the quality of life at T1.

	Univariate model			Multivariate model		
Variables	PR	95% CI	p-value	PR	95% CI	P-value
Age	1.01	0.97 - 1.05	0.539			
Sex						
Female - male	1.45	0.85 - 2.53	0.185			
Upper Retainer						
Absent	1					
Fixed	1.02	0.59 - 1.74	0.955			
Lower Retainer						
Absent	1					
Fixed	1.44	0.75 - 3.06	0.311			
Self-perception of change in teeth position						
Yes - No	1.71	1.02 - 2.90	0.05*	1.57	0.93 - 2.65	0.1
Fracture/debonding of retainer						
Yes - No	1.89	1.01 - 3.27	0.028*	1.76	1.01 - 3.07	0.05*

CI = Confidence Interval, *p < 0.05, PR = Prevalence Ratio.

At T1, four years after T0, the mean of the OHIP-14 increased significantly to 8.31 (SD=7.57, *p* = 0.014) as shown in Tables 1 and 4.

**Table 4:** Analysis of variance for the change in OHIP from T0 to T1 (repeated measures) for the groups with and without recurrence and with or without retainer fracture or debonding.

		Mean Difference	p-valor Tukey
OHIP - T0	OHIP - T1	-2.9	0.014*
Relapse NO	Relapse YES	-2.09	0.231
Fracture / Debonding NO	Fracture / Debonding YES	-4.91	0.038*
Fracture / Debonding NO	no retainer	0.21	0.996
Fracture / Debonding YES	no retainer	5.11	0.139

* p <0,05.

**Figure 1:**
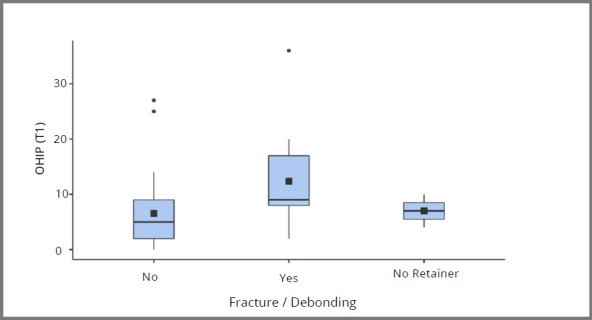
OHIP-14 T1 boxplot according to self-perception of retainer fracture and/or debonding.

ANOVA for repeated measures evaluated the changes in OHIP-14 from T0 to T1. The OHIP-14 increased significantly from T0 to T1, on average 2.9 (*p* = 0.014), and was not significantly different between patients who did or did not experience any relapse (*p* = 0.231). However, patients with relapse reported a value of OHIP-14 2.09 times greater than those who did not observe changes in the position of the teeth. Reports of retention problems, such as debonding or fracture, were associated with a worse quality of life, since patients who reported a fracture or debonding reported a higher OHIP score, on average 4.91 times, when compared to those who did not report it (Table 4, Fig 1).

## DISCUSSION

Obtaining a normal occlusion after orthodontic treatment is associated with a higher level of quality of life related to oral health,^2^ ^,^ ^24^ which justifies the findings of a low OHIP-14 score, increasing significantly four years later . This increase, although statistically significant, does not seem to depend on the continued use of a retainer. However, a large variability observed in the OHIP-14 values between the groups was not detected, given the sample size of the present study.

All patients at T0 had normal treated occlusion, indicating stability after orthodontic treatment. On the other hand, since patient dissatisfaction is associated with the use of retainers,^7^ the results of the present study corroborate the literature by stating that the use of fixed upper retainers was negatively associated with quality of life, since in the short term, the use of this retainer worsens the quality of life, compared to patients without an upper retainer.

Bonding failures of the upper retainer are a frequent problem during the retention phase. In a previous study, it was reported that a total of 58.2% of all patients had retainer failures and the chances were higher for type 3x3.^25^ In the present study, 28.9% of the patients presented with debonding or fracture in the first three years after placement of the retainer, however, they did not report a negative influence on the quality of life.

Considering that patient satisfaction is only slightly associated with treatment stability,^6^ it seems reasonable that the self-perception of changes in the position of the teeth, considered an indicator of possible relapses, did not have an impact on quality of life.

In the long-term evaluation at T1, the use of a fixed upper retainer had no impact on quality of life, which may represent the patient’s adaptation to the use of the appliance. What initially caused discomfort and impact on quality of life over time becomes a habit and seems to no longer represent a nuisance. However, it seems clear that if this retainer undergoes a debonding or fracture, this event will have a significant negative impact on quality of life. 

The presence of a fixed upper retainer causes an initial negative impact on the patient’s quality of life after completing orthodontic treatment. In the long term, our hypothesis was confirmed, since 4 years after the initial evaluation, the patient’s quality of life improved and they could adapt to prolonged use by performing routine checks. The worsening of OHRQoL, observed in the long term, is not associated with the presence of the retainer itself, but with the occurrence of debonding or fracture. This study emphasizes the need for continuous follow-up of orthodontic patients during the retention period. It can be mentioned that during the COVID-19 pandemic, patients were absent from their routine visits, which could be a contributing factor to this debonding or fracture, thus affecting the patient’s quality of life.

Although orthodontists have differing opinions regarding the type of upper retainer, there seems to be a greater preference for the use of fixed retainers in the lower arch.^11^ ^,^ ^17^ Previously published studies suggest that fixed retainers offer a greater benefit in preserving mandibular incisor alignment in the long term.^26^ ^,^ ^27^ Our findings suggest that the use of lower fixed retainers was not associated with oral health-related quality of life at T0 and T1. However, this study did not include any patients using a removable retainer, which is considered to be a more uncomfortable device when compared to a fixed lower retainer.^7^

The use of maxillary fixed retainers does not appear to cause significant negative effects on periodontal health, despite a slight increase in plaque accumulation.^26^ ^,^ ^28^ Previous studies have reported a higher rate of breakage or debonding associated with the use of fixed upper retainers.^28^ In the present study, 28.9% of the patients perceived a break in the retainer, associated with a worse quality of life in the long term (*p*= 0.05). However, its use is recommended since, in the long term, fixed maxillary and mandibular retainers are effective in maintaining intercanine width^29^, being necessary to use retainers permanently to avoid relapses in the distant future.^18^ ^,^ ^19^

The assessment of the impact of changes from T0 to T1 showed that patients who used upper and/or lower retainers and stopped using them after a while showed no difference in their quality of life, as well as those who still use the retainer. These results differ from the literature that reports that the use of retainers can cause more discomfort,^7^ although studies have highlighted that fixed maxillary retainers may not make a long-term difference in terms of maxillary incisor alignment.^15^ ^,^ ^30^ Thus, it seems clear that prolonged use of upper retainers needs to be discussed. It is important to emphasize that the assessment of the quality of life can be influenced by several variables, including characteristics related to malocclusion that can influence the retention protocol, dental conditions involving episodes of pain, caries, periodontal problems, in addition to economic, social factors, cultural and implications of the COVID-19 pandemic.^31^

It is important to consider that the quality of life related to oral health tends to undergo negative changes during the isolation period of the COVID-19 pandemic^32^ due to the decrease in the clinical control of the retainers, which can present fracture or debonding and, consequently, the movement in the position of teeth. Randomized studies evaluating the long-term stability and impact of the COVID-19 pandemic on quality of life-related to the use of retainers are needed.

Regarding the limitations of the present study, the absence of an evaluation of the psychological profile at T0 and T1 must be considered. This is because the psychological profile can be associated with a high degree of demand or expectations that are far from reality^33^ and consequently compromise patients’ perception of orthodontic treatment and quality of life.

In addition, there is no questionnaire developed and validated specifically to assess the impact of orthodontic retainers on quality of life. Although the OHIP-14 questionnaire is closer to an ideal assessment, its use may have influenced the results of this study. Due to restrictions related to the COVID-19 pandemic, an online questionnaire was used to assess patients’ self-perception. However, the application of the questionnaire in this format did not interfere negatively in the results of the study, since the questions and answers are the same as the questionnaire applied on printed paper in the initial evaluation.^34^

Removable orthodontic retainers are associated with greater patient discomfort;^17^ however, this study was performed only on patients with fixed retainers. Patients with removable retainers who completed the questionnaire at T0 were not included, as in the second assessment (T1) most were no longer using retainers.

Finally, the lack of clinical assessment of T1 patients suppresses information relevant to this study, such as a possible association between periodontal status and the quality of life reported by patients. Factors associated with COVID-19 made clinical assessment impossible at T1 and may have contributed to the increase in the OHIP value in the sample examined.

## CONCLUSIONS

After the completion of orthodontic treatment, the presence of a fixed upper retainer have an initial negative impact on the quality of life of the orthodontic patient. The impact of the presence of a retainer is mitigated in the long term. The worsening in OHRQoL, observed in the long term, is not associated with the presence of the retainer itself but with the occurrence of debonding or fracture. This study emphasizes the need for continuous follow-up of orthodontic patients during the retention period.
